# Inbreeding depression by environment interactions in a free-living mammal population

**DOI:** 10.1038/hdy.2016.100

**Published:** 2016-11-23

**Authors:** J M Pemberton, P E Ellis, J G Pilkington, C Bérénos

**Affiliations:** 1 Institute of Evolutionary Biology, School of Biological Sciences, University of Edinburgh, Edinburgh, UK

## Abstract

Experimental studies often find that inbreeding depression is more severe in harsh environments, but the few studies of *in situ* wild populations available to date rarely find strong support for this effect. We investigated evidence for inbreeding depression by environment interactions in nine traits in the individually monitored Soay sheep population of St Kilda, using genomic inbreeding coefficients based on 37 037 single-nucleotide polymorphism loci, and population density as an axis of environmental variation. All traits showed variation with population density and all traits showed some evidence for depression because of either an individual's own inbreeding or maternal inbreeding. However, only six traits showed evidence for an interaction in the expected direction, and only two interactions were statistically significant. We identify three possible reasons why wild population studies may generally fail to find strong support for interactions between inbreeding depression and environmental variation compared with experimental studies. First, for species with biparental inbreeding only, the amount of observed inbreeding in natural populations is generally low compared with that used in experimental studies. Second, it is possible that experimental studies sometimes actually impose higher levels of stress than organisms experience in the wild. Third, some purging of the deleterious recessive alleles that underpin interaction effects may occur in the wild.

## Introduction

Reduced fitness is a near-universal consequence of inbreeding in diploid organisms, and we now know much about why it occurs (Charlesworth and Charlesworth, 1987; Keller and Waller, 2002; Charlesworth and Willis, 2009). In brief, recessive or partially recessive deleterious alleles, which circulate at low frequency, relatively unexposed to selection in a population, have increased probability of being homozygous and hence expressed in inbred individuals; a second and probably less common mechanism is that any loci that show heterozygous advantage (i.e., overdominance) are more likely to be homozygous in inbred individuals. The inbreeding load of a population is a function of the accumulation of deleterious recessive alleles and the extent to which inbreeding has exposed them to selection and hence purging, and load therefore varies between species and populations within species (Keller and Waller, 2002).

The fitness of an individual is a function of its intrinsic quality (including the expression of any deleterious recessive alleles) and extrinsic factors, for example, the environment it finds itself in. Whether inbreeding *interacts* with environmental heterogeneity to affect fitness has interested biologists for many years, for two main reasons. First, population and conservation biologists are interested in whether the factors they consider (for example, environmental conditions and inbreeding) act additively or interact, potentially adversely, so that inbreeding is exacerbated under poor environmental conditions (Armbruster and Reed, 2005; Fox and Reed, 2011; Reed *et al.*, 2012) and contribute to population extinction (Liao and Reed, 2009). Second, evolutionary geneticists are interested in what such interactions reveal about the genetics of inbreeding—for example, are the effects because of changing expression of the same alleles, or are new alleles being expressed under specific environmental circumstances (Armbruster and Reed, 2005; Fox and Reed, 2011; Reed *et al.*, 2012).

Taken together, experimental studies in animals and plants show clear evidence for inbreeding depression by environment interactions (ID × E). A meta-analysis of 34 studies concluded that 76% of cases showed an increase of inbreeding depression in more stressful environment (48% found significant increases), overall amounting to a 69% increase in inbreeding depression in the more stressful environment (Armbruster and Reed, 2005). Another meta-analysis concluded that inbreeding depression scales linearly with the magnitude of stress (defined as the relative survival of outbred individuals in stressful and benign environments), such that a population suffers one additional lethal equivalent for each 30% reduction in fitness of outbreds induced by the environment (Fox and Reed 2011), whereas a recent experiment on *Drosophila* suggests nonlinear effects at high stress and inbreeding levels (Schou *et al.*, 2015). Both meta-analyses emphasise heterogeneity in the data for different species and lineages within species, reflecting the high element of chance in the segregation of deleterious recessives among populations.

The experimental studies reported in the above meta-analyses usually manipulate inbreeding so that individuals fall into two or more classes: an outbred class and one or more defined inbred class, and most also manipulate the environment into stress categories. In addition, a few studies have compared fitness in inbred and outbred individuals in captive versus natural or seminatural environments and generally report quite strong interactions (see, for example, Jimenez *et al.*, 1994; Meagher *et al.*, 2000; Joron and Brakefield, 2003; Enders and Nunney, 2012), and a comparison of inbreeding depression in zoo populations and wild populations (using largely different species in each category) concluded that inbreeding depression is more severe in the wild than in captivity (Crnokrak and Roff, 1999). Observational studies conducted wholly within actual wild populations (which are few in number, see below) were either deliberately excluded from the recent meta-analyses (Armbruster and Reed, 2005) or form only a small proportion of the studies included (Fox and Reed, 2011), although the need for such studies is often alluded to in discussions (Crnokrak and Roff, 1999; Armbruster and Reed, 2005; Reed *et al.*, 2012). Observational, correlational studies have the clear disadvantage that they cannot prove causality. However, there are at least two ways in which they can contribute to the field to its advantage.

A first advantage of observational studies of ID × E interactions in the wild is that the levels of inbreeding under study are realistic for the study population. Experimental studies commonly compare outbred (*F*=0) with inbred (typically *F*=0.25 for animals and *F*=0.5 for plants). Whereas selfing is common in many plants and a surprising number of animals (for example, gastropods), and hence *F*=0.5 is a realistic level of inbreeding for such species, *F*=0.25, as obtained under parent–offspring or full-sib mating, is generally rare in wild populations of animals that only have biparental inbreeding (see below). Instead, such populations typically exhibit a continuum of much lower inbreeding coefficients and hence the ID × E experimental literature does not represent routine occurrence but instead what might happen following catastrophic population decline. Put another way, the standardisation of inbreeding depression to, for example, *F*=0.25 or *F*=1 using the coefficient of inbreeding depression or lethal equivalents (see, for example, Crnokrak and Roff, 1999; Fox and Reed, 2011), which is desirable because it allows direct comparison of inbreeding load between studies and populations, nevertheless ignores the fact that absolute levels of inbreeding are often low in natural populations with biparental inbreeding.

A second advantage of *in situ* studies is that the environmental heterogeneity experienced by a population is generally that which it has evolved to deal with. Experimental studies can easily manipulate environmental variation beyond the natural range likely to be experienced by the study organism in nature. In this regard the idea of a standardised measure of the stress imposed by environmental conditions (Fox and Reed, 2011) that can be applied to both experimental and wild systems is useful. A separate point is that experimental studies, at least in principle, can manipulate the environment on axes of heterogeneity that are not normally met by the species in the wild or what it would normally be able to avoid. Given the potential role of purging, this again makes wild population studies desirable.

**Figure 1:**
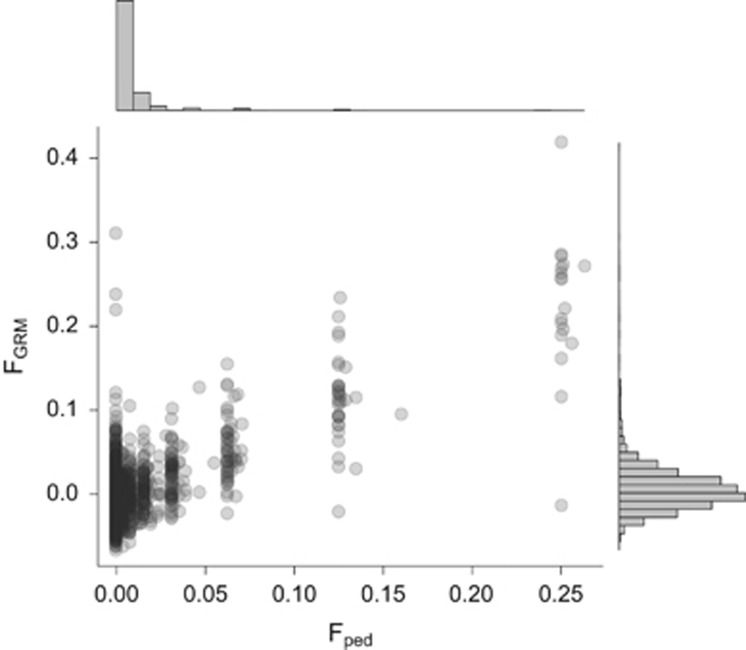
The correlation between pedigree inbreeding coefficients (*F*_ped_) and genomic inbreeding (*F*_GRM_) for 3765 genotyped sheep that have at least two parents and one maternal grandparent known (*y*=0.899*x*−0.005, *r*^2^=0.42).

There are practical reasons why studies of ID × E in the wild are rare. First, fitness data on individuals need to be available for many individuals experiencing a range of environmental conditions in time or space. Second, estimates of inbreeding coefficients for the same individuals must be available. Estimating pedigree inbreeding coefficients with precision is hard because deep, accurate pedigrees are difficult to obtain (Pemberton, 2008). Although a social pedigree may be a good guide to parentage in some bird species, extra-pair paternity is common and can substantially alter estimates of fitness, inbreeding coefficients and hence inbreeding depression (Reid *et al.*, 2014), and in polygynous species, behaviour is rarely a good guide to paternity. Across breeding systems, genetic parentage inference thus ranges from desirable to essential. Genetic parentage assignment requires adequate sampling of offspring and candidates that can be challenging. Immigrant individuals are another problem: their ancestry is unknown and hence they are commonly assumed to be unrelated to the rest of the study population which may be untrue and will bias the inbreeding coefficients of their descendants downwards. Molecular markers offer an alternative route to estimating the inbreeding status of an individual, but until recently, the number of markers available in natural population studies (typically panels of 10–20 microsatellites) had only low power to detect inbreeding depression via heterozygosity–fitness correlations (Balloux *et al.*, 2004; Slate *et al.*, 2004; Chapman *et al.*, 2009). More recently, however, it has been shown that large panels of single-nucleotide polymorphism (SNP) markers generate estimates of individual inbreeding that offer three advantages in natural populations (Hoffman *et al.*, 2014; Bérénos *et al.*, 2016; Huisman *et al.*, 2016). A big advantage is that the inbreeding status of an individual is judged from its own DNA sample and is not contingent on samples from and known ancestry of other individuals in the way that a pedigree estimate is. A second, related, advantage is that genomic inbreeding tends to be normally distributed, and hence it is more convenient statistically (Figure 1). Finally, genomic inbreeding estimators capture variation in inbreeding around the expected pedigree inbreeding coefficient because of chromosome assortment and recombination (Hill and Weir, 2011, 2012), although the importance of this extra precision in predicting inbreeding depression has yet to be formally demonstrated.

Despite the difficulties of studying ID × E interactions in the wild, we have located 10 published studies of 11 natural populations that have investigated inbreeding by environment interactions to date, the results of which are summarised in Table 1. These studies tested 96 potential ID × E interactions but only 12 were statistically significant, of which 4 involve rainfall in different intervals during song sparrow incubation, that is, they consider a suite of overlapping environmental variables (Marr *et al.*, 2006). This ‘success rate' in finding ID × E effects stands in sharp contrast to the results of the meta-analyses of the mainly experimental studies reported above. Furthermore, investigations of ID × E in wild populations might be affected by the file drawer problem, that is, null results for ID × E analyses may be underreported, perhaps because of the concerns about the precision of inbreeding estimators reported above.

In this study we investigate ID × E in an individually monitored free-living mammal population, the Soay sheep of St Kilda. The rate of inbreeding in this population is generally low, for example, just 0.5% of individuals are inbred at *F*=0.25 (Figure 1; more details in the Discussion). Nevertheless, we have previously found inbreeding depression in three juvenile body size traits and four of six fitness components using a genomic estimator of inbreeding (*F*_GRM_) based on 37 037 SNP loci genotyped in a large sample of sheep (Bérénos *et al.*, 2016). Specifically, we found statistically significant depression associated with an individual's own *F*_GRM_ in 4-month weight and hindleg length, and we found inbreeding depression associated with the *mother's F*_GRM_ in offspring birth weight and 4-month weight. Among fitness components, we found inbreeding depression in adult female annual survival and in male first year survival, adult male annual survival and adult male annual breeding success.

On St Kilda, conditions fluctuate dramatically from year to year, with population density a key determinant of variation in both phenotypic traits and fitness components. High density depresses birth weights, lamb body size, fecundity and winter survival (Clutton-Brock *et al.*, 1992, 2004a; Coulson *et al.*, 2001, 2008; Forchhammer *et al.*, 2001). Natural selection on phenotypic traits is also consistently stronger during high density winters (Milner *et al.*, 1999; Morrissey *et al.*, 2012). In the analysis of inbreeding depression described above (Bérénos *et al.*, 2016), year of birth and/or year of measurement was included as a random effect in the models to account for variation in conditions between years. Here, we extend these models by fitting population density as a fixed effect and then investigate ID × E by fitting an interaction term for *F*_GRM_ × population density. We predicted that inbreeding depression and density would interact in our study system, and that high density would exacerbate the effect of inbreeding on juvenile body size and fitness components.

## Materials and methods

### Study system and morphological data collection

**Figure 2:**
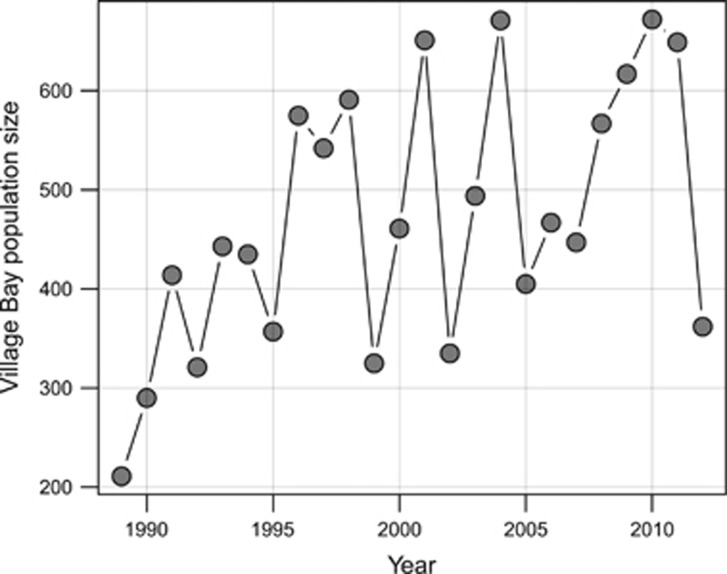
Temporal dynamics of the population size of sheep resident in Village Bay between 1989 and 2012.

The Soay sheep is a primitive breed that lives in an unmanaged state on the islands of Hirta and Soay in the St Kilda archipelago, NW Scotland (Clutton-Brock *et al.*, 2004b). The breed is descended from the first sheep brought to the British Isles during the Bronze Age, but it also experienced an admixture event with the Dunface sheep breed in the nineteenth century (Feulner *et al.*, 2013). Sheep resident in the Village Bay area of Hirta, where approximately one-third of the sheep inhabiting the island are found, have been the subject of a long-term individual-based study since 1985. Most individuals (ca. 95%) are captured, ear-tagged and weighed within a few days of birth in April. Every August, ~60% of resident sheep are captured and several morphometric measures are taken, including hindleg and body weight. Winter mortality is monitored, with the peak of mortality occurring at the end of winter, and ca. 80% of all deceased sheep are found. As there are no predators or competing herbivores on the island, mortality is because of malnutrition exacerbated by gastrointestinal parasite infection (Gulland *et al.*, 1993). Overwinter mortality varies dramatically from year to year, depending on the population density entering the winter, winter weather conditions and the proportion of vulnerable individuals (for example, young and old) in the population (Coulson *et al.*, 2001, 2008). Density was here estimated from regular censuses as the number of sheep living in Village Bay on 1 October each year, excluding males that were only present for a short period during the rut. Village Bay population size is strongly correlated with the whole island population size (*r*^2^=0.90). Over the years considered here (1989–2012, including the year before the 1990 cohort was born), the Village Bay population size has fluctuated between 211 and 672 (Figure 2).

### Parentage inference and pedigree construction

Parentage was inferred through a combination of observational field data and molecular markers for maternal links, and using molecular markers only for paternal links (Johnston *et al.*, 2013; Bérénos *et al.*, 2014). Molecular parentage assignments were predominantly (for 4371 individuals) obtained using 315 polymorphic and unlinked SNP markers selected from the SNP chip data described below and assigned with 100% confidence in the R package *MasterBayes* (Hadfield *et al.*, 2006). In some cases SNP genotypes were not available for either lamb or candidate fathers and paternity was assigned using 14–18 polymorphic microsatellite markers (for a total of 222 lambs, assignment with confidence >95% in *MasterBayes* (Morrissey *et al.*, 2012)). This enabled the construction of a pedigree with a maximum depth of 10 generations and consisting of 6740 individuals, of which 6336 were nonfounders. More details about pedigree construction and pedigree summary statistics can be found in Johnston *et al.* (2013) and Bérénos *et al.* (2014).

### Genotype data

Individuals were genotyped using the Ovine SNP50 BeadChip (Illumina, San Diego, CA, USA) using an iScan instrument at the Wellcome Trust Clinical Research Facility Genetics Core (Edinburgh, UK). Quality control was performed in PLINK (Purcell *et al.*, 2007). Individuals with call rate >95% were retained, and loci with minor allele frequency <0.01, call rate <99% or which strongly deviated from Hardy–Weinberg equilibrium at *P*<1e−05 were discarded. Note that just 580 SNPs were discarded because of deviation from Hardy–Weinberg equilibrium, indicating no pervasive deviation from Hardy–Weinberg equilibrium across all SNPs that would be indicative of intense inbreeding; most likely, these loci contain various genotyping errors. A total of 37 037 autosomal SNP loci remained after quality control. Median spacing between SNPs was 50.2 kb, and neighbouring SNPs were generally in high linkage disequilibrium (mean *r*^2^=0.3). For more details on data acquisition and quality control see Bérénos *et al.* (2014).

### Inbreeding estimator

For each individual we calculated the genomic inbreeding estimator *F*_GRM_. We have previously shown that this estimator is more strongly associated with a range of fitness components and aspects of juvenile body size than either pedigree inbreeding or several other genomic estimators (Bérénos *et al.*, 2016). *F*_GRM_ is a genome-wide estimate of inbreeding that is a weighted average across all loci (Fhat3 in Yang *et al.*, 2011) and was calculated in the GCTA software (Yang *et al.*, 2011). This estimator gives more weight to homozygotes of the minor allele than to homozygotes of the major allele at each locus, and has a lower sampling variance than other homozygosity-based single SNP measures (Yang *et al.*, 2011). *F*_GRM_ for each SNP *i* and individual *j* is calculated as follows:


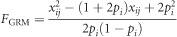


where *x* is the number of copies of the reference allele (0, 1 or 2) and *p* is the population-wide allele frequency of the reference allele. *F*_GRM_ is strongly correlated with *F*_ped_ (Figure 1).

### Estimation of inbreeding depression by environment effects

For information on the measurement of birth weight, 4-month hindleg and 4-month weight, see Beraldi *et al.* (2007). We included individuals born between 1990 and 2012, the latter being the last cohort for which genomic data are currently available. We analysed these continuous traits using linear mixed models (so-called animal models) in ASREML-r (Gilmore *et al.*, 2009) as previous research has demonstrated that in Soay sheep they harbour significant variation because of additive genetic variation, maternal additive genetic variation and maternal environment (Bérénos *et al.*, 2014) and (more broadly) that if both additive genetic and inbreeding effects are present, estimating *V*_a_ and inbreeding depression simultaneously yields conservative estimates of each (Wolak and Keller, 2014). The models included lambs of both sexes as sex differences in these traits are small and easily dealt with by a fixed effect of sex. Random terms included an additive genetic effect, a year of birth effect, a maternal additive genetic effect and a maternal environmental effect representing remaining effects due to the identity of the mother that have also all previously been shown to contribute significantly to trait variance (Bérénos *et al.*, 2014). To estimate additive and maternal genetic effects, relatedness matrices were calculated using the pedigree. A comprehensive list of fixed effects fitted in the various models, known from previous analyses of juvenile body size traits (Bérénos *et al.*, 2014), is shown in Table 2. To estimate the effects of population density (*E*) and inbreeding depression (*ID*) and the interaction between them, two parallel models were run. Note that in this population, both maternal and offspring inbreeding have a potential effect on juvenile body size, and therefore both effects were fitted, following Bérénos *et al.* (2016). First, a model was run that included *E* (population density) and *F*_GRM_ (both maternal and individual) as main effects only (model 1). We examined whether the density on 1 October before birth (i.e., overwinter density while the lamb was *in utero*) or density on 1 October after birth (i.e., density during the postnatal growth phase) had the strongest effect on juvenile body size by running two separate models, each containing either this year's or the previous year's population density. After establishing which population density had the strongest effect, we then ran the exact same model, but including the interaction term between population density and *F*_GRM_, and the interaction was fitted both for maternal and individual *F*_GRM_ (model 2). Statistical significance of fixed effects was assessed by Wald *F*-statistics.

We next examined the effects of *E* and *F*_GRM_ on first winter survival, annual survival (for individuals aged 1 or older) and annual breeding success for individuals born between 1990 and 2012. As males and females have very different survival rates (males survive less well than females) and reproductive scheduling (females produce 0–2 lambs every year, whereas males produce 0–22 per year, with non-zero values mainly occurring in older males) we modelled the fitness components separately for each sex. First winter survival was a binary response variable describing whether an individual that was alive on 1 November survived past 1 May in the year following birth. For each sheep year *j*, adult annual survival was a binary response variable describing whether or not an individual survived past 1 May in year *j* + 1, and annual breeding success was defined as the number of offspring born in year *j*. All fitness components were analysed using Generalised Linear Mixed Models (GLMM) with a Bayesian approach using Markov chain Monte Carlo (MCMC) algorithms in the R package *MCMCglmm* (Hadfield, 2010). We analysed the fitness components in a Bayesian framework because they have nonnormal distributions: a categorical (binomial) error distribution was used for first year overwinter survival and adult annual survival and a Poisson error distribution was used for annual breeding success. We did not use an animal model approach for the fitness components because previous research suggests negligible additive genetic variance for these traits as well as for lifetime breeding success (Morrissey *et al.*, 2012; Johnston *et al.*, 2013). For models analysing fitness components, in addition to population density and individual and maternal *F*_GRM_, fixed effects were chosen based on Johnston *et al.* (2013) and Bérénos *et al.* (2015) and subsequent exploratory analyses (for a full list of fixed effects fitted, please see Table 2). Birth weight was included in the models of first winter survival as we were interested in the effects of inbreeding on fitness over and above the effects it may have through reduced birth weight (Jones *et al.*, 2005). Birth weight was corrected by taking the residuals from a model containing a significant third-order polynomial that best described the relationship between age at capture (in days, only including individuals that were captured 10 days post birth or earlier) and weight at capture (in kg). Random effects included are shown in Table 2. As with the juvenile body size traits, two parallel models were run. First, a model was run that included population density, individual and maternal *F*_GRM_ as main effects only (model 1). Second, a model was run that included the interaction between population density and *F*_GRM_ (model 2). The interaction term was fitted for both individual and maternal inbreeding for first year overwinter survival, but was only fitted for individual inbreeding in adult fitness components as maternal inbreeding had little effect on adult fitness components. For the fitness measures the population density used was the density at 1 October just before the start of the mating season and before overwinter mortality and parturition occurred. To accommodate differences in model complexity and data structure between models, MCMC chain length varied between the models, but all chains were run for at least 1 000 000 iterations with a burn-in phase of at least 200 000 iterations, and at least 2000 independent samples were taken from the posterior at equally spaced intervals. Priors were specified for random effects, such that the total phenotypic variance was divided equally between the random effects fitted and for survival residual variance was fixed at one. Exploratory analyses suggested that model estimates are not dependent on the priors used. Convergence was assessed by visual inspection of the traces and was deemed acceptable if autocorrelation between successive samples was below 0.05. Results are presented as posterior modes of the sampled iterations and the 95% credibility interval. Significance of effect sizes can be assumed if the 95% credibility interval does not overlap with zero.

## Results

### Main effects of population density and inbreeding (model 1)

Rising population density depressed all the traits studied and was highly significant in eight of the nine traits, the exception being female adult breeding success, for which *P*=0.098 (Table 3). Population density on 1 October before birth was the best predictor for the juvenile body size traits (tests not shown).

In the juvenile body size traits, inbreeding depression associated with a sheep's own *F*_GRM_ was detected in 4-month hindleg and 4-month weight, and inbreeding depression associated with a mother's *F*_GRM_ was detected in offspring birth weight and offspring 4-month weight (Table 3), recapitulating our previous results (Bérénos *et al.*, 2016) with the exception that the association between a mother's *F*_GRM_ and her offspring's 4-month weight was weaker (*P*=0.053). In the fitness components there were no associations with maternal *F*_GRM_ (as before) but there were highly significant negative associations with a sheep's own *F*_GRM_ in adult annual female survival, male first winter survival, adult male annual survival and adult male annual breeding success. As before, there was also a trend for negative association between *F*_GRM_ and female first winter survival (at *P*=0.066). The single appreciable change because of fitting density as a fixed effect was that adult female annual breeding success, which showed a trend for inbreeding depression in our previous modelling (*P*=0.067), was significant in the model including density (*P*=0.025), meaning that all six fitness components (three components in each sex) show some evidence for inbreeding depression (Table 3).

In summary, all the traits studied were negatively associated with both inbreeding coefficient and density and thus provide an appropriate scenario in which to search for ID × E effects.

### Interactions between population density and inbreeding (model 2)

**Figure 3:**
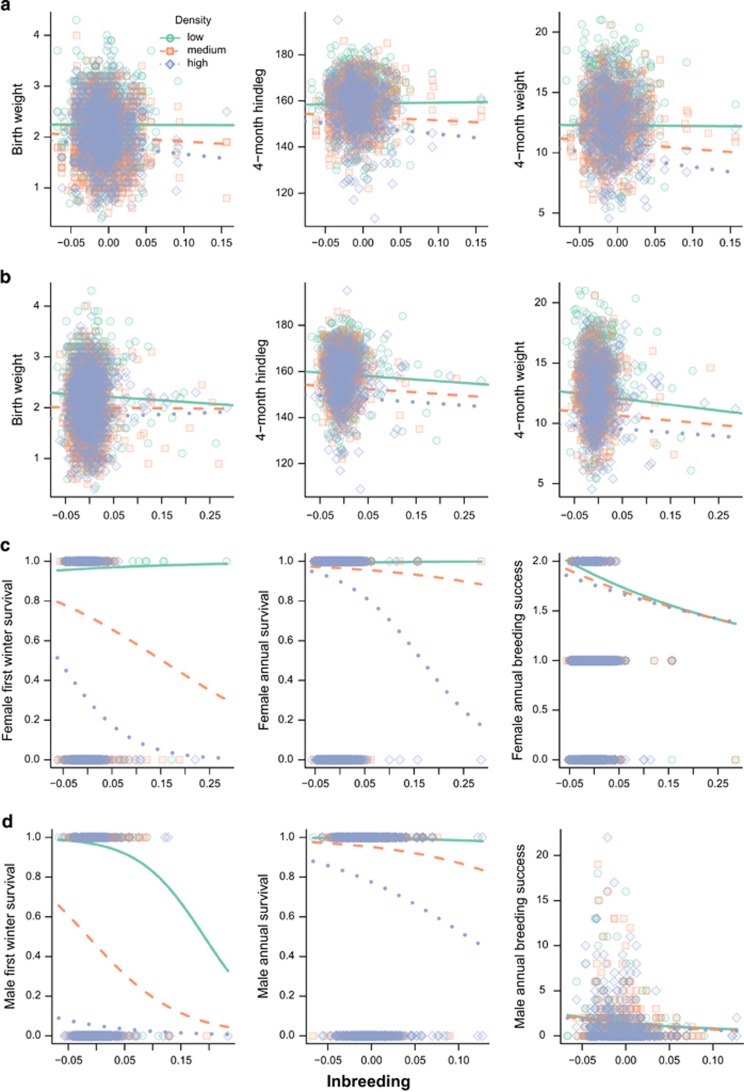
The effect of inbreeding (*F*_GRM_) on juvenile weight (**a** and **b**) and three fitness components in females (**c**) and males (**d**). Row (**a**) shows the effects of maternal inbreeding depression and all other rows show the effects of inbreeding depression expressed through the focal individual. Symbols representing the raw data are colour coded based on population density by dividing the population densities into three sets of equal size, and the fitted lines represent predicted values for the midpoints of each group (low: 311, medium: 481, high: 611). Note that the only formally significant interactions were in birth weight (interaction between maternal inbreeding and density, *P*=0.045; see Table 3) and in adult female annual survival (interaction between inbreeding and density, *P*=0.02; see Table 3) and neither would survive Bonferroni correction. A full color version of this figure is available at the *Heredity* journal online.

All three juvenile body size traits showed a consistent interaction pattern between maternal *F*_GRM_ and previous year's population density, in which inbreeding depression was more severe following a high density year (Table 3 and Figure 3). However, only one of these interactions, for birth weight, was formally statistically significant (*P*=0.045), whereas the other two were just not significant (4-month hindleg *P*=0.059; 4-month weight *P*=0.069). In addition, there was an interaction between an individual's own *F*_GRM_ and previous population density at *P*=0.071 for birth weight, in which inbreeding depression was more severe after a low population year (i.e., opposite to expectation), but no such interaction was found for 4-month hindleg or 4-month weight (Table 3 and Figure 3).

In the fitness components, the results of fitting ID × E terms were different between the traits. For three of the four survival traits (female first winter survival and adult annual survival in both sexes), there was a consistent pattern in which inbreeding depression became more intense with rising density (Figure 3), but only the interaction for adult female annual survival was statistically significant (*P*=0.02); for female first winter survival it was just below significance at *P*=0.06 and adult male annual survival was not significant (*P*=0.924; Table 3). Formally, male first winter survival showed a nonsignificant (*P*=0.492) interaction opposite to that expected, with inbreeding depression less severe at high density (Table 3). Inspection of Figure 3 shows that this pattern is somewhat driven by very high mortality regardless of inbreeding at high density and that some ID × E in the expected direction may be present at medium–low densities. Annual breeding success showed no sign of ID × E interaction in either sex, either when plotted (Figure 3) or statistically (Table 3).

In summary, of the nine traits studied, we conducted 14 tests for ID × E (considering both focal and maternal inbreeding). Six tests on six different traits show a pattern of intensifying inbreeding depression with increasing population density, but only two of these patterns were statistically significant at *P*<0.05 and they would not survive Bonferroni correction for 14 tests. Two tests on two different traits showed a pattern of ID × E in the opposite direction to that expected (that is, inbreeding depression less severe at high density) but neither was statistically significant.

## Discussion

In this study we have investigated ID × E in nine traits (three morphometric and six fitness) in a large wild animal data set with an accurate genomic estimator of individual inbreeding. In all nine traits there was a strong negative effect of population density and there was some evidence of inbreeding depression. Six traits showed some evidence of ID × E in the expected direction (Figure 3), but overall the effects were weak and barely significant. Despite using a superior estimator of individual inbreeding, an environmental variable with appreciable effects and substantial sample sizes, our results are thus consistent with those of the studies reported in Table 1: in nature, ID × E effects are hard to detect.

One obvious reason for our failure to detect convincing interaction terms may be a lack of power in the data set. It is noticeable that the two traits providing strongest evidence for ID × E both have the largest sample sizes in their group. Thus, birth weight (2810 observations on 697 mothers) is the most data-rich trait among the juvenile body size traits (Table 3) and female adult annual survival, along with female adult annual breeding success (both 3229 observations on 640 females), is the most data-rich trait in the fitness components group (Table 3). However, sample sizes vary widely among ID × E studies in wild animal studies (Table 3) and there is no discernible association between sample size and success in finding ID × E at this stage.

Of course, the possibility that the data set analysed here may not have the power to detect statistically significant ID × E effects is a symptom of the fact that any ID × E effect sizes are small. There are three potential explanations for such small effects. First, the amount of inbreeding may be too low to enable detection of ID × E and, second, perhaps the effect of population density on most study traits was in practice too modest to enable detection of ID × E. Third, perhaps the alleles that confer more inbreeding depression at high density have been purged from the population.

The variance in inbreeding coefficients sets a ceiling on detectable inbreeding depression. In Soay sheep, despite the population being an island isolate, rates of inbreeding are low and variation in inbreeding is also low. Based on minimal pedigree requirements (both parents and at least one maternal grandparent known), there are 811/3816 individuals (22%) with non-zero pedigree inbreeding coefficients (*F*_ped_), of which just 18/3816 (0.5%) are inbred at *F*_ped_=0.25 (which results from parent–offspring or full-sib mating) (Bérénos *et al.*, 2016). With 78% of individuals having *F*_ped_=0, both the mean and variance of *F*_ped_ are low (0.005±0.00053). The genomic estimator *F*_GRM_ is on a slightly different scale to *F*_ped_ in that mean *F*_GRM_ is centred on zero and particularly outbred individuals take small negative values of *F*_GRM_. With this measure and using the same pedigree criteria, 1616 individuals/3765 (43%) have *F*_GRM_ >0 but only 12/3765 (0.3%) have *F*_GRM_ >0.23 and the variance is again low (0.00090) (Bérénos *et al.*, 2016). Note that the two estimators of inbreeding are correlated across individuals (Figure 1). Thus, although using *F*_GRM_ approximately doubles the proportion of individuals with non-zero inbreeding coefficients, and provides rich detail on the inbreeding status of each individual, most of the variation detected is because of individuals with small deviations above and below zero *F*_GRM_ (Figure 1), and the variance is less than doubled compared with *F*_ped_. In a parallel analysis in red deer, variance in inbreeding increased from 0.00084 in *F*_ped_ to 0.00114 in *F*_GRM_ (Huisman *et al.*, 2016) that is, by ∼40%, but remains overall low. It is easy to see why the detection of ID × E is difficult in Soay sheep when the majority of inbreeding coefficients are low, the relatively few high values are spread over the >20 years of the study and density takes extreme values in only some years.

Comparable estimates for mean and variance in inbreeding in other wild populations are hard to obtain from the current literature, but strongly suggest that levels of inbreeding in species with only biparental inbreeding are low. For example, Table 1 lists a measure of inbreeding that was available for each study population investigated for ID × E. The method of reporting is highly heterogeneous and is most frequently a proportion of individuals with *F*_ped_ ⩾0.125 or 0.25 within a sample defined by a certain amount of pedigree information. Inspection of Table 1 shows that even in island populations, the rate of inbreeding is low, with all studies reporting only a few percentage of individuals born with *F*_ped_ ⩾0.125 or 0.25. An exception is the reintroduced population of the endangered Stewart Island robins with 15.9% of individuals having *F*_ped_ ⩾0.125 (Laws *et al.*, 2010). Collated estimates of mean and variance in inbreeding for a number of wild, endangered and captive populations similarly suggest low levels of inbreeding for wild populations (Grueber *et al.*, 2011).

When considering rates of inbreeding it must also be borne in mind that inbreeding depression in survival causes temporal changes in mean and variance in inbreeding with age. The estimates quoted for Soay sheep and red deer above, for other species in Table 1 and in other wild populations (Grueber *et al.*, 2011) are commonly for individuals that were known to exist and/or reached sampling age. If there has been mortality associated with inbreeding before sampling, then the quoted figures may underestimate the actual amount of inbreeding undertaken by breeders. By the same token, if there is mortality associated with inbreeding *after* sampling, so-called intragenerational purging (Enders and Nunney 2016), then this will restrict further the variance in inbreeding available for studying traits measured later in life. Thus, the fact that there is strong inbreeding depression in male first winter survival in Soay sheep (Table 3) will reduce the opportunity to detect inbreeding depression in male adult annual survival and male annual breeding success because inbred males have already been removed from the distribution.

For comparative purposes, it would be highly desirable for the reporting of rates of inbreeding to be standardised. A problem is that the amount of inbreeding based on a pedigree varies strongly with the depth of known pedigree and with data selection decisions (Marshall *et al.*, 2002; Bérénos *et al.*, 2016; Huisman *et al.*, 2016). Even when, as in many of the studies in Table 1 and in the Soay sheep (above), minimum data selection criteria are used, researchers do not generally truncate the depth of pedigree by removing ancestors, and hence the amount of pedigree information available per individual can be extremely variable. Such data selection issues are not so problematic for F_GRM_, although there may still be a need to consider issues such as discriminating locally born from immigrant individuals. Overall, as more pedigree- and genome-based studies of inbreeding depression come on stream, we urge researchers to clearly state the stage at which inbreeding rate is being measured, the pedigree criteria for including an individual in an inbreeding study and standard statistics for rates of inbreeding, that is, mean and variance.

A second reason for our failure to detect ID × E may be that density dependence as a main effect was weak in some traits. Of the nine traits studied, annual breeding success has the weakest main effects of density (model 1 estimate <<−0.001 in females and −0.002 in males; Table 3) that may explain why neither female nor male annual breeding success showed any sign of ID × E (Figure 3). The weak density dependence in female annual breeding success is due to the fact that the majority of females produce one lamb per year, and variance in this trait is mainly because of yearlings that reproduce (rather than not) and some older females having twins, both of which are density dependent (Clutton-Brock *et al.*, 2004a). However, even in a model of yearling female reproduction, in which density dependence was strong, there was no ID × E interaction (model not shown), possibly for reasons of low sample size (*N*=640). Density dependence of male annual breeding success has been demonstrated before in Soay sheep but is again weak in some sectors of the male population (lambs and yearlings) (Pemberton *et al.*, 1999). This trait also has a modest sample size compared with some traits in the current analysis (1251 observations on 446 individuals).

Inspection of the four survival plots in Figure 3 suggests another reason why ID × E may be hard to detect in our (and possibly other wild) studies: the effects of density are overwhelming. At low density, the model predictions are for essentially 100% survival in female first winter survival, adult female annual survival and adult male annual survival. At high density, the model predictions are for ∼0% first winter survival of males, and we do indeed observe such extremes. In these sectors of the population there is little opportunity for inbreeding depression. Although a lack of inbreeding depression under benign conditions might be expected if good environmental conditions can compensate for being inbred, the idea that extremely harsh environmental conditions can also mask inbreeding depression (Armbruster and Reed 2005) is perhaps not so widely realised. It has previously been pointed out that researchers studying ID × E should consider the opportunity for selection (Waller *et al.*, 2008). Here, we suggest a refinement to this line of thinking by suggesting that prior knowledge of the effects of environmental conditions on traits could be used to make and test precise predictions about the kind of environmental conditions likely to generate variance in the trait. For example, we could have tested for ID × E in male first winter survival only in medium and low density years.

Recent experimental studies of ID × E have standardised on a specific measure of environmental stress in order to allow comparison across inbreeding levels, environmental variables and studies, and it is of interest to consider this stress metric in the context of Soay sheep traits and density. Stress is measured as the effect of the environmental variable on the survival of an outbred control group, and in recent studies has been manipulated to range from 0 to 0.6 or 0.8 (meaning 40–20% survival of outbreds) (Fox and Reed, 2011; Enders and Nunney, 2012; Schou *et al.*, 2015). An impression of the stress imposed by different density values in outbred Soay sheep can be gained by inspection of the left-hand ends of the lines depicting three different densities in the survival traits in Figure 3. Outbred lambs of both sexes have close to 100% survival at low density, whereas at high density it is typical for only 10% of outbred male lambs and 50% of outbred female lambs to survive, indicating that stress ranges up to at least 0.90 for males and 0.50 for this life-history component in males and females, respectively, and goes even higher at the highest densities. However, for adult survival the lines for different densities are much closer together indicating much lower levels of stress. The latter is also true of all the other traits, were we to apply a similar approach to estimating stress. However, despite the stress of different densities being greatest for first winter survival, and despite some evidence for inbreeding depression in this trait (especially in males), the interaction term was not significant in either sex (Table 3). Conversely, the trait providing the strongest evidence for ID × E in our study was female annual survival, a trait showing minimal stress because of density. This is a puzzling pattern, suggesting as it does that stress measured in outbreds is not necessarily a good predictor of when ID × E may occur, a topic worth further investigation.

A third and final explanation for the difficulty of detecting ID × E in free-living populations may be that the alleles responsible for it have been purged from the population. Experimental work in *Drosophila* suggests that ID × E effects occur because of the expression of different deleterious recessive alleles under different conditions, such that purging is likely to be environment specific (Bijlsma *et al.*, 1999), a prediction supported by a direct test (Swindell and Bouzat, 2006). Soay sheep have existed unmanaged for millennia in a state of density dependence, in which selection on most traits is stronger at higher density (Milner *et al.*, 1999; Morrissey *et al.*, 2012). Recessive and partially recessive alleles that contribute to reduced performance at elevated density are especially likely to have been exposed to selection. This hypothesis thus suggests that natural populations are perhaps particularly unlikely to show ID × E interactions because the axes of environmental variation that can be measured are ones to which populations have been exposed over evolutionary time and in which purging of deleterious recessives may have occurred. Conversely, experimental studies are more likely to test values or axes of environmental variation to which the study population has not previously been exposed. The above-noted contrast between male first winter survival (inbreeding depression, high stress but no ID × E) and female adult annual survival (inbreeding depression, low stress and ID × E) is interesting in this context: purging will be a lot more efficient in lambs than adults because the stress is higher and it is occurring earlier in the life history.

The Soay sheep population of Hirta, St Kilda, is an island isolate with a maximum observed population size of 2200 and an estimated *N*_e_ of 194 (Kijas *et al.*, 2009); it makes a reasonable model for a population of conservation concern, and indeed as a rare sheep breed under a unique management regime it *is* a population of conservation concern. However, neither inbreeding nor ID × E interactions seem to be a current threat to population persistence in Soay sheep: despite the effects of inbreeding and ID × E documented here and in Bérénos *et al.* (2016), the population trend is currently increasing (Figure 2). Soay sheep population size is strictly regulated by the overwinter carrying capacity of the island and the erratic population dynamics are strongly determined by the population size entering the winter, overwinter weather and the sex/age structure of the population: in years when high density, adverse weather and a vulnerable population coincide, mortality can be very high (Coulson *et al.*, 2001). Although inbred individuals die in these crashes, the population rapidly builds up again after each crash (Figure 1).

Interactions between inbreeding depression and environment have been incorporated into models of population persistence to suggest that they are an important contributor to the risk of population extinction (Liao and Reed, 2009). Our results and those of others (Table 1) suggest a more nuanced approach to this issue may be appropriate. Certainly, sudden, intense inbreeding is likely to have an adverse effect on a population, as is environmental deterioration. Whether the two interact in an important way will depend on the intensity of the inbreeding and may depend on critical features of the environmental variables such as whether they are novel to the population or not.

## Data archiving

Data available from the Dryad Digital Repository: http://dx.doi.org/10.5061/dryad.8v010.

## Figures and Tables

**Table 1:** Published studies testing for an interaction between inbreeding depression and environmental variation in free-living populations

*Species*	*Method for estimating inbreeding*	*Amount of inbreeding in pop*	*Whose inbreeding is being studied?*	*Trait*	*Sample size*	*Inbreeding depression main effect detected?*	*Environmental variable(s) tested*	*Environmental variable(s) that interacted with ID*	*Effect on inbreeding depression*	*Reference*
Soay sheep, *Ovis aries*	Microsatellite heterozygosity	8/899=6.1% of individuals with all 4 grandparents known have *F*_ped_ ⩾0.125.	Individual adults	Fecal egg count	398	Y	Year	Year	ID more severe in high density years	(Coltman *et al.*, 1999)
Cactus finch, *Geospiza scandens*	Social pedigree *F*	3.3% Of individuals with all 4 grandparents known have *F*_ped_ ⩾0.25	Individual	Survival from banding to age 1 year	120	Y	Annual rainfall^a^, density of *G. scandens*, density of *G. fortis* and all interactions between	Annual rainfall	ID only apparent when rainfall low	(Keller *et al.*, 2002)
			Individual	Adult annual survival		Y	Annual rainfall, density of *G. scandens*, density of *G. fortis* and all interactions between	Annual rainfall × density of *G. scandens*	ID more severe when rainfall low and *G. scandens* density high	
			Individual	Adult annual probability of breeding		Y	Annual rainfall, density of *G. scandens*, density of *G. fortis* and all interactions between	None	NA	
Medium ground finch, *Geospiza Fortis*	Social pedigree *F*	0.8% Of individuals with all 4 grandparents known have *F*_ped_ ⩾⩾0.25.	Individual	Survival from banding to age 1 year	364	N	Annual rainfall, density of *G. fortis*, density of *G. scandens* and all 2-way interactions between	None	NA	(Keller *et al.*, 2002)
			Individual	Adult annual survival		N	Annual rainfall, density of *G. fortis*, density of *G. scandens* and all interactions between	None	NA	
			Individual	Adult annual probability of breeding		Y	Annual rainfall, density of *G. fortis*, density of *G. scandens* and all interactions between	None	NA	
Collared flycatcher, *Ficedula albicollis*	Social pedigree *F*	16/2107=0.8% clutches with sufficient pedigree to detect *F*_ped_=0.125 had *F*_ped_ ⩾0.25.	Clutch	Clutch size	2088	N	Average body condition in year of birth	None	NA	(Kruuk *et al.*, 2002)
			Clutch	Hatching success	2066	Y	Average body condition in year of birth	None	NA	
			Clutch	Fledging success	2065	N	Average body condition in year of birth	None	NA	
			Clutch	Fledgling tarsus length	1971	Y	Average body condition in year of birth	None	NA	
			Clutch	Fledgling body condition	1966	N	Average body condition in year of birth	None	NA	
			Clutch	Juvenile survival	1760	Y	Average body condition in year of birth	None	NA	
			Clutch	Recruitment per nest	1764	Y	Average body condition in year of birth	None	NA	
Seychelles warbler, *Acrocephalus sechellensis*	Microsatellite heterozygosity	5% Of 119 offspring estimated to be due to matings between first-order relatives	Female parent	Offspring survival	119	N	Breeding season	Breeding season	In a low-survival breeding season, offspring survival was correlated with maternal heterozygosity, but not in other seasons.	(Richardson *et al.*, 2004)
Song sparrow, *Melospiza melodia*	Social pedigree *F*	<10% Of birds in male mating success analysis had *F* ⩾0.125	Adult male	Male mating success	680 Obs on 262 males	Y	Number of males, males per female	None	NA	(Marr *et al.*, 2006)
			Male social parent	Offspring survival from 12 to 24 days	650 Obs on 179 males	Y	Rainfall in 2-day, 3-day and 4-day rainiest interval^b^	None	NA	
			Female parent	Laying date	371 Obs on 166 females	Y	Average daily temperature measured across six different pre-laying time intervals	None (second best model showed an interaction with temperature)	NA (second best model suggests reduced difference between inbred and outbred females under cool temperatures)	
			Female parent	Hatching success	640 Obs on 155 females	Y	Average daily rainfall across four time intervals during incubation	Average daily rainfall in all the intervals studied	Increased ID in rainy conditions.	
Great tit *Parus major*	Social pedigree *F*	58/4523=1.3% of broods with both parents and one grandparent known have *F*_ped_ ⩾0.125	Breeding events (=clutches)	Number of offspring recruited	Around 4500 clutches	Y	Population density, local oak density, female age, male age, local population density, distance from forest edge, caterpillar lag, yearly fledging mass, winter beech mast, yearly recruitment quality (all grouped into above and below average)	Yearly fledging mass	Increased ID in years of low fledging mass	(Szulkin and Sheldon, 2007)
Stewart island robin, *Petroica australis rakiura*	Social pedigree *F* (with some genetic support)	29/182=15.9% of broods have *F*_ped_ ⩾0.125. All four grandparents known for 96% of broods.	Brood, female parent, male parent	Hatching success	182 Broods	N	Min temperature during nesting period, rainfall during nesting period,^c^ habitat type.	None	NA	(Laws *et al.*, 2010)
			Brood, female parent, male parent	Fledging success		N	Min temperature during nesting period, rainfall during nesting period, habitat type.	None	NA	
			Brood, female parent, male parent	Recruitment success		N	Min temperature during nesting period, rainfall during nesting period, habitat typez	None	NA	
Red deer, *Cervus elaphus*	Genetic pedigree *F*	36/1848=2% Of individuals with both parents and at least one grandparent known inbred at F_ped_ ⩾0.125	Calf and mother	Birth date	2515 Calves from 602 mothers	Offspring F: N, maternal F: N	Year of birth	None	NA	(Walling *et al.*, 2011)
				Birth weight	1664 Calves from 487 mothers	Offspring F: Y, maternal F: N	Year of birth	None	NA	
				First year survival	1593 Calves from 463 mothers	Offspring F: Y, maternal F: N	Year of birth	None	NA	
				First winter survival	1400 Calves from 443 mothers	Offspring F: Y, maternal F: N	Year of birth	None	NA	
Meerkats *Suricata suricatta*	Genetic pedigree *F*	71/1583=4.4% of pups had *F*_ped_ ⩾0.125. All four grandparents known for 97% of pups.	Pup	Emergence mass	422	Y	Number of lactators Number of helpers	Number of lactators	⩽4 Lactators increased emergence mass, but ⩾5 lactators depressed that of inbred pups	(Nielsen *et al.*, 2012)
				Hindfoot length	219	Y	Number of lactators Number of helpers Season Rainfall	None	NA	
				Growth until independence	523	Y	Number of lactators Number of helpers Rainfall	Number of lactators	More lactators were associated with slower growth of inbreds.	

Abbreviations: ID, inbreeding depression; N, no; NA, not available; Obs, observations; Y, yes.

a In the Galapagos, rainfall is positively associated with food production.

b On Mandarte Island, rainfall during the breeding season is associated with breeding failure.

c On Stewart Island, rainfall during the breeding season probably depresses brood survival.

**Table 2:** List of fixed and random effects fitted in the models in addition to the effects of inbreeding and density

		*Fixed effects*						*Random effects*					
*Trait*	*Sex (male or female)*	*Litter size (0 or 1)*	*Maternal age (years, quadratic)*	*Age (years, quadratic)*	*Age at capture*	*Birth day*	*Adjusted birth weight (kg)*	*Year of birth*	*Sheep year*	*Sheep ID*	*Maternal ID*	*Additive genetic effect*	*Maternal genetic effect*
Birth weight	x	x	x		Days (factor)	x		x				x	x
Hindleg	x	x	x		Days	x		x				x	x
Weight	x	x	x		Days	x		x				x	x
First winter survival		x	x	x			x	x			x		
Annual survival				x				x	x		x		
Annual breeding success				x				x	x	x	x		

Abbreviation: ID, inbreeding depression.

**Table 3:** Parameter estimates showing the effects of inbreeding, maternal inbreeding, population density and the interaction between inbreeding and population density

*Trait*	*Sample size*^a^	*Parameter*	*Main effects only*		*ID* × *E interaction fitted*	
			*Estimate*	P	*Estimate*	P
Birth weight	2810	Inbreeding	−0.13 (0.234)	0.579	−1.666 (0.887)	0.597
	697	Maternal inbreeding	−0.865 (0.419)	**0.04**	1.469 (1.24)	**0.037**
		Density	−0.001 (0)	**<0.001**	−0.001 (0)	**<0.001**
		Density:inbreeding			0.003 (0.002)	0.071
		Density:maternal inbreeding			−0.005 (0.002)	**0.045**
4-Month hindleg	1665	Inbreeding	−14.616 (6.39)	**0.022**	−15.922 (24.861)	**0.025**
	582	Maternal inbreeding	−13.988 (9.411)	0.138	42.509 (31.402)	0.136
		Density	−0.032 (0.003)	**<0.001**	−0.033 (0.003)	**<0.001**
		Density:inbreeding			0.003 (0.051)	0.947
		Density:maternal inbreeding			−0.122 (0.064)	0.059
4-Month weight	1661	Inbreeding	−3.881 (1.453)	**0.008**	−6.794 (5.666)	**0.009**
	582	Maternal inbreeding	−4.541 (2.339)	0.053	7.994 (7.299)	0.05
		Density	−0.008 (0.001)	**<0.001**	−0.009 (0.001)	**<0.001**
		Density:inbreeding			0.006 (0.012)	0.586
		Density:maternal inbreeding			−0.027 (0.015)	0.069
Female first winter survival	1219	Inbreeding	−6.535 (−13.138, 0.717)	0.066	24.112 (−7.898, 60.656)	0.153
	531	Maternal inbreeding	2.385 (−6.207, 11.396)	0.595	−9.781 (−50.485, 30.445)	0.626
		Density	−0.0142 (−0.0208, −0.0075)	**<0.001**	−0.0144 (−0.0214, −0.008)	**<0.001**
		Density:inbreeding			−0.064 (−0.136, 0.003)	0.06
		Density:maternal inbreeding			0.024 (−0.053, 0.103)	0.549
Adult female annual survival	640 (3229)	Inbreeding	−11.284 (−17.221, −5.071)	**<0.001**	27.889 (−6.84, 63.545)	0.115
	371	Maternal inbreeding	−2.363 (−10.13, 4.628)	0.515	−1.904 (−9.046, 5.685)	0.609
		Density	−0.0088 (−0.0142, −0.004)	**0.002**	−0.0092 (−0.0148, −0.0041)	**0.002**
		Density:inbreeding			−0.068 (−0.131, −0.009)	**0.02**
Female annual breeding success	640 (3229)	Inbreeding	−1.915 (−3.573, −0.221)	**0.025**	−3.191 (−10.274, 4.56)	0.396
	371	Maternal inbreeding	−1.002 (−2.826, 0.689)	0.261	−0.987 (−2.763, 0.779)	0.279
		Density	−5e−04 (−0.001, 1e−04)	0.074	−5e−04 (−0.001, 1e−04)	0.098
		Density:inbreeding			0.003 (−0.012, 0.017)	0.73
Male first winter survival	1035	Inbreeding	−11.37 (−18.878, −2.655)	**0.008**	−26.53 (−68.359, 18.52)	0.24
	507	Maternal inbreeding	−7.681 (−17.709, 1.954)	0.121	21.771 (−24.959, 67.008)	0.369
		Density	−0.0211 (−0.0325, −0.0113)	**<0.001**	−0.0217 (−0.0325, −0.0105)	**<0.001**
		Density:inbreeding			0.03 (−0.054, 0.112)	0.492
		Density:maternal inbreeding			−0.06 (−0.158, 0.028)	0.202
Adult male annual survival	446 (1251)	Inbreeding	−11.088 (−19.592, −2.965)	**0.01**	−8.427 (−59.569, 41.515)	0.748
	295	Maternal inbreeding	4.223 (−5.628, 13.636)	0.398	4.198 (−5.607, 14.134)	0.389
		Density	−0.0138 (−0.0236, −0.0046))	**0.003**	−0.0139 (−0.0232, −0.0045)	**0.001**
		Density:inbreeding			−0.005 (−0.089, 0.084)	0.924
Male annual breeding success	446 (1251)	Inbreeding	−8.753 (−14.812, −2.725)	**0.003**	−10.635 (−29.217, 5.978)	0.23
	295	Maternal inbreeding	2.752 (−4.501, 9.211)	0.424	2.765 (−4.313, 9.555)	0.427
		Density	−0.0024 (−0.0042, −5e−04)	**0.014**	−0.0024 (−0.0043, −6e−04)	**0.015**
		Density:inbreeding			0.004 (−0.031, 0.036)	0.839

Abbreviation: ID × E, inbreeding depression by environment interaction.

Density is for 1 October before birth for the juvenile body size traits and for 1 October before winter and parturition for the fitness components. Parameter estimates show s.e. for juvenile body size traits and 95% credibility intervals for fitness components. Note that slope estimates for density are for each additional sheep in the population which varied between 211 and 672 across the study years. Statistically significant *P* values are shown in bold.

a Numbers give the number of unique individuals and the number of observations is shown in parentheses.
